# Supplementary material for: Mating system of Datura inoxia: association between selfing rates and herkogamy within populations
Source: PeerJ. 2021 Mar 19;9:e10698. doi: 10.7717/peerj.10698 (PMC7983856; doi:10.7717/peerj.10698)
Supplement: Supplemental Information 1 [file peerj-09-10698-s001.docx]

**Supplemental information: PCR Protocols**

All PCR were performed in 25uL reactions in a Maxygene termal cycler ( Axygen- Foster City, CA). The PCR procedure was: 5 min at 94°C, 35 cycles of 1 min at 94°C, 1 min 47-58.5 °C, 1 min at 72°C, and a final extension for 5 min 72°C. Each reaction contained 2 μL of genomic DNA; 2.5 uL 10X buffer RED Taq ReadyMix PCR Reaction Mix (with 20 mmol/L TrisHCl pH 8.3); 100 mmol/L KCl; 0.75 uL MgCl 2; 0.002% gelatin; 0.4 mmol/L dNTP mix (dATP, dCTP, dGTP, dTTP); stabilizers; 1 unit/μL of TaqDNA polymerase) (Sigma-Aldrich; St. Louis, Missouri, USA), and 15.75 uL of pure water. PCR products were visualized via 1 % agarose gel electrophoresis and it were run on ABI 3100 Genetic analyser (Applied Biosystems, Foster City, CA) using 500 Liz size ladders. Fragment analyses were carried out with Gene Marker software v.2.4.0 (SoftGenetics, LLC, State College, PA, USA).
