# Supplementary material for: Mating system of Datura inoxia: association between selfing rates and herkogamy within populations
Source: PeerJ. 2021 Mar 19;9:e10698. doi: 10.7717/peerj.10698 (PMC7983856; doi:10.7717/peerj.10698)
Supplement: Supplemental Information 2 [file peerj-09-10698-s002.docx]

| **A. Primary selfing rate (*r*)** |  |  |  |  |
| --- | --- | --- | --- | --- |
| Source | *df* | *MS* | *F* | *P* |
| Populations | 1 | 1.204 | 12.1 | 0.001** |
| Herkogamy | 1 | 0.835 | 8.4 | 0.005** |
| Error | 53 | 0.099 |  |  |
| **B. Inbreeding coefficient (*F*)** |  |  |  |  |
| Source | *df* | *MS* | *F* | *P* |
| Populations | 1 | 0.246 | 2.002 | 0.163 |
| Herkogamy | 1 | 0.026 | 0.210 | 0.648 |
| Error | 53 | 0.123 |  |  |
| **C. Inbreeding at equilibrium (*F_e_*)** |  |  |  |  |
| Source | *df* | *MS* | *F* | *P* |
| Populations | 1 | 1.491 | 15.489 | 0.000*** |
| Herkogamy | 1 | 0.745 | 7.736 | 0.008* |
| Error | 53 | 0.096 |  |  |
| **D. *F_e_* - *F*** |  |  |  |  |
| Source | *df* | *MS* | *F* | *P* |
| Populations | 1 | 2.945 | 21.215 | 0.000*** |
| Herkogamy | 1 | 0.493 | 3.549 | 0.065 |
| Error | 53 | 0.139 |  |  |

* *P* < 0.05, ** *P* ≤ 0.005, *** *P* < 0.0005 indicate significant association between variables.
