# Supplementary material for: Mating system of Datura inoxia: association between selfing rates and herkogamy within populations
Source: PeerJ. 2021 Mar 19;9:e10698. doi: 10.7717/peerj.10698 (PMC7983856; doi:10.7717/peerj.10698)
Supplement: Supplemental Information 3 [file peerj-09-10698-s003.docx]

| **Model** | **g.l.** | **AIC** |
| --- | --- | --- |
| r ~ Herk, link= "cloglog" | 3 | -13.461 |
| r ~ Herk, link= "logit" | 3 | -13.426 |
| r ~ Herk, link= "log" | 3 | -13.381 |
